# Fear of COVID-19 Predicts Depression, Anxiety and Post-Traumatic Stress Disorders in Patients with Implantable Cardioverter Defibrillators and Is Mediated by Positive and Negative Affects—A Cross-Sectional Study

**DOI:** 10.3390/jcm12216884

**Published:** 2023-10-31

**Authors:** Marc Dörner, Roland von Känel, Aju P. Pazhenkottil, Rahel Altwegg, Ladina Nager, Veronica Attanasio, Lisa Guth, Sina Zirngast, Anna Menzi, Mary Princip, Claudia Hackl-Zuccarella

**Affiliations:** 1 Department of Consultation-Liaison-Psychiatry and Psychosomatic Medicine, University Hospital Zurich, University of Zurich, 8091 Zurich, Switzerland; roland.vonkaenel@usz.ch (R.v.K.); aju.pazhenkottil@usz.ch (A.P.P.); rahel.altwegg@usz.ch (R.A.); ladina.nager@usz.ch (L.N.); veronica.attanasio@usz.ch (V.A.); lisa.guth@usz.ch (L.G.); sina.zirngast@usz.ch (S.Z.); mary.princip@usz.ch (M.P.); claudia.hackl-zuccarella@usz.ch (C.H.-Z.); 2 German Center for Neurodegenerative Diseases (DZNE) within the Helmholtz Association, 39120 Magdeburg, Germany

**Keywords:** implantable cardioverter defibrillator, fear of COVID-19, COVID-19 pandemic, depression, anxiety, post-traumatic stress disorder, social support, mental health

## Abstract

The COVID-19 pandemic affected both the physical and mental health of the general population. People with cardiac diseases seem to be particularly vulnerable to the implications of the pandemic. However, studies on the mental health impact of the COVID-19 pandemic on people with implantable cardioverter defibrillator (ICDs) are lacking. Thus, we aimed to explore the level of fear of COVID-19 and the prevalence of anxiety, depression and post-traumatic stress disorder (PTSD) in ICD patients. Furthermore, we aimed to identify novel predictors for anxiety, depression and PTSD, including COVID-19-related variables, and to assess whether positive affects (PAs) and negative affects (NAs) mediate the relationship between the level of fear of COVID-19 and anxiety, depression and PTSD, respectively. The data of 363 patients with ICDS who had been prospectively included in this study between 2020 and 2023, were analyzed. Potential predictors for anxiety, depression, and PTSD were identified using logistic regression. To identify indirect mediating effects of PAs and NAs, we applied the PROCESS regression path analysis modeling tool. The prevalence of anxiety was 9.19%, of depression 10.85%, and of PTSD 12.99%. Being unemployed was the strongest predictor for anxiety (OR = 10.39) and depression (OR = 6.54). Younger age predicted anxiety (OR = 0.95) and PTSD (OR = 0.92). Receiving low social support was associated with anxiety (OR = 0.91), depression (OR = 0.88) and PTSD (OR = 0.91). Patients with a history of COVID-19 (OR = 3.58) and those who did not feel well-informed about COVID-19 (OR = 0.29) were more likely to be depressed. Higher levels of fear of COVID-19 predicted anxiety (OR = 1.10), depression (OR = 1.12) and PTSD (OR = 1.14). The relationship between fear of COVID-19 and anxiety or depression was fully mediated by PAs and NAs, while NAs partially mediated the relationship between fear of COVID-19 and PTSD. Vulnerable subgroups of ICD patients may need additional psychological and educational interventions due to fear of COVID-19, anxiety, depression and PTSD during the pandemic.

## 1. Introduction

The coronavirus disease 2019 (COVID-19) pandemic, caused by the spread of the severe acute respiratory syndrome coronavirus 2 (SARS-CoV-2), has emerged as a major cause of morbidity and mortality worldwide. Consequently, the global health care system faced unparalleled challenges [1].

Preventive measures, such as quarantine, lockdowns and social distancing, effectively mitigated the spread of the SARS-CoV-2. However, these measures also led to a high psychosocial impact on society, causing social isolation, significant changes in lifestyle, financial losses and uncertainty about the future [2,3]. Patients with chronic cardiac diseases who are in need of frequent contact with health care providers, were particularly advised to reduce medical and social contacts to prevent COVID-19 infections since COVID-19 can affect the cardiovascular system and may result in myocardial injury, arrhythmias and sudden cardiac death [4].

An Implantable Cardioverter Defibrillator (ICD) treats and prevents those life-threatening conditions by anti-tachycardia pacing or shocks. ICD implantation and ICD therapy (shocks) are associated with worse emotional functioning and reduced quality of life compared to controls [5]. Indeed, a recent meta-analysis indicated a high prevalence of post-traumatic stress disorder (PTSD, 12%), depression (15%) and anxiety (22%) in ICD patients [6]. Moreover, those psychiatric symptoms and disorders were found to predict mortality in patients with an ICD [7].

Unsurprisingly, prior studies demonstrated that the COVID-19 pandemic affects both physical and mental health, leading to increased levels of stress, anxiety, fear, post-traumatic stress symptoms (PTSS) and depression in the general population, and facilitating or exacerbating psychiatric disorders [8,9]. Furthermore, fear of COVID-19 might cause delayed healthcare access, anxiety, depression and even suicide [10,11]. Hence, research on the impact of the COVID-19 pandemic on mental health is urgently required to identify detrimental psychological problems, and to implement adequate interventions to prevent or diminish those problems.

However, until now, no data are available regarding the mental health impact of the COVID-19 pandemic on ICD patients. This knowledge could also prove valuable for the clinical management of patients with an ICD during potential future pandemics. Since this patient group is particularly vulnerable to COVID-19 [4], mental health problems indicated to predict mortality in those patients [7], and the COVID-19 pandemic led to increased levels of anxiety, PTSS and depression in the general population [8,9], we aimed to evaluate the prevalence of anxiety, depression and PTSD, and the level of fear of COVID-19 in patients with ICDS spanning the whole course of the COVID-19 pandemic (2020–2023), and to identify predictors of anxiety, depression and PTSD in ICD patients, which might be linked to those symptoms or disorders, including fear of COVID-19 [11], ICD shocks [5] and a history of COVID-19 [11]. We hypothesized that (1) patients with an ICD show a higher prevalence of anxiety, depression and PTSD during the pandemic compared to findings before the pandemic [6], as was demonstrated for the general population and patients with psychiatric disorders [8,9]. Furthermore, we hypothesized that (2) COVID-19-related variables, such as fear of and a history of COVID-19 [11], predict anxiety, depression and PTSD in ICD patients. Additionally, we hypothesized that (3) negative (NAs) and positive affects (PAs) mediate the relationship between fear of COVID-19 and anxiety, depression and PTSD, respectively. We assume that the individual level of fear of COVID-19 leads to a change in NA, reflected by unpleasable engagement and subjective distress, and PAs, representing pleasurable engagement with the environment. Then, the change in PAs and NAs might result in anxiety, depression and PTSD according to the tripartite model, an influential conceptualization of anxiety and depression [12,13].

## 2. Materials and Methods

### 2.1. Design and Sample

**Figure 1:**
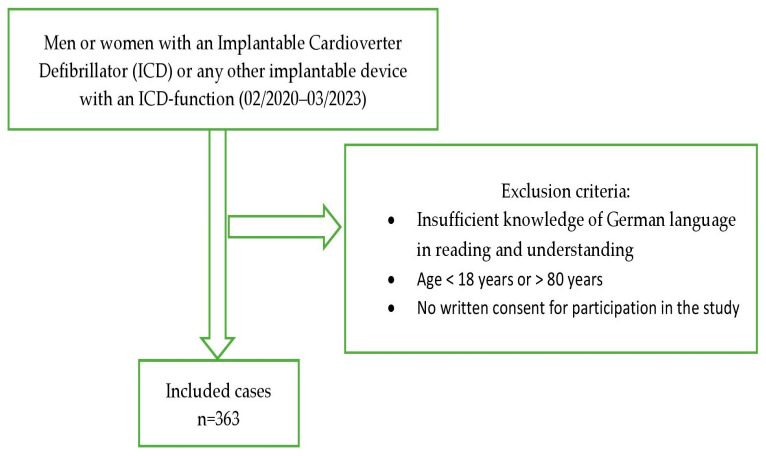
Process of selecting the study sample. *n* = number.

In this national, single-centre cross-sectional study, we prospectively included *n* = 363 participants with an implantable device with an ICD function who were recruited at their half-yearly routine check-up at the Cardiac Arrhythmia Division (Department of Cardiology) at the University Hospital Zurich between February 2020 and March 2023. Patients younger than 18 years or older than 80 years, and those lacking German language skills, were excluded from the study. Figure 1 depicts the procedure for selecting the study sample. Participants were asked to fill out various self-report questionnaires regarding psychometric variables, sociodemographics, medical-related variables and ICD concerns. The study was approved by the Cantonal Ethics Committee of Zurich (no. 2019-01948; 12/2019) and all participants provided written consent.

### 2.2. Instruments

#### 2.2.1. Fear of COVID-19 Scale (FCV-19S)

To measure the severity of stress response to the COVID-19 pandemic and the general level of fear of COVID-19, which may have an important impact on ICD patients (i.e., more shocks due to the stress and higher levels of depression, anxiety, and PTSS), we used the Fear of COVID-19 Scale (FCV-19S). The FCV-19S is a seven-item unidimensional scale, which is translated and validated in many countries. Answers are given on a five-item Likert-type scale (1 = strongly disagree to 5 = strongly agree). Total scores are calculated by adding up each item score (range 7 to 35 points). Higher scores indicate greater fear of COVID-19 [14].

#### 2.2.2. Measurements of Depression, Anxiety and Post-Traumatic Stress Disorder (PTSD)

We used the Patient Health Questionnaire-8 (PHQ-8) for the diagnosis and assessment of depression. It includes eight items from the diagnostic criteria for major depressive disorder (MDD) and has been utilized in various studies as a screening test for the general population as well as for specific disease populations. Each item of the PHQ-8 is scored from 0 (absent) to 3 points (severe). Prior studies suggested a cut-off score of 10 or higher as an indicator of MDD [15].

The Generalized Anxiety Disorder-7 (GAD-7) is an effective and well-established questionnaire for anxiety disorders. Although developed for the identification of GAD, it is also used to detect other anxiety disorders, such as panic disorder or social anxiety. The GAD-7 has seven items with response categories ranging from 0 (not at all) to 3 (nearly every day). A cut-off score of 10 or higher was found to have optimal sensitivity and specificity values for identifying GAD [16]. According to a systematic review, the GAD-7 showed the best performance characteristics for the detection of GAD compared to other screening tools [17].

The Post-Traumatic Stress Diagnostic Scale (PDS, according to Diagnostic and Statistical Manual of Mental Disorders-4) is a brief and reliable self-report measure of PTSD [18]. The PDS is conducted in clinical and research settings, and using a four-point scale, respondents are asked to rate 17 items comprising the cardinal symptoms of PTSD during the last 30 days [19]. Various studies identified a cut-off score of 14 or higher to have optimal sensitivity and specificity for the detection of PTSD [20,21].

#### 2.2.3. Measurements of Positive and Negative Affects

In order to measure the level of PAs and NAs in our study sample over the last twelve months, participants applied the Positive and Negative Affect Schedule (PANAS), a 20-item self-report questionnaire. The PANAS consists of two 10-item mood scales, a PA and NA item scale. PA items include active, interested, excited, strong, inspired, proud, enthusiastic, alert, determined and attentive. NA items consist of distressed, upset, guilty, scared, hostile, irritable, ashamed, nervous, jittery and afraid. Answers are given on a five-point scale (1 = very slightly or not at all to 5 = very much), and both PA and NA scores range from 10 to 50 points [12].

#### 2.2.4. ENRICHD Social Support Instrument (ESSI)

Since previous studies suggested an important role of social support in the outcomes of ICD patients [22], we also applied the ENRICHD Social Support Instrument (ESSI), a 7-item self-report questionnaire, which evaluates social support. The first six items consist of a 5-point Likert scale (1 to 5), and the seventh item is a yes or no question (4 points for yes and 2 for no). The total score ranges from 8 to 34 points, and higher scores indicate higher levels of social support [23].

### 2.3. Data Analysis

For statistical analysis, we used IBM SPSSS Statistics, version 29 (Chicago, IL, USA). To describe patient characteristics, such as age, sex, educational status, a history of COVID-19, number of ICD shocks and psychometric scores, we calculated mean and median scores, standard deviation, and relative and absolute distributions. Normally distributed continuous variables were compared with an unpaired *t*-test and one-way ANOVA, and chi-square and Fisher’s exact test, where appropriate, were used to compare normally and non-normally distributed categorical variables. To exclude issues of multicollinearity, collinearity statistics were applied. In order to identify variables, which predict the presence of depression, anxiety or PTSD, we conducted a binary logistic regression with a PHQ-8 score ≥ 10 (yes or no), GAD-7 score ≥10 (yes or no) and a PDS score ≥ 14 (yes or no) as the dependent variables, respectively. Independent variables, such as age, sex, educational status, and other covariates that are related to depression, anxiety or PTSD according to existing literature, were also entered into the regression models: fear of COVID-19 [11] as measured by the FCV-19S [14], a serologically confirmed history of COVID-19 (yes or no) [11], the number of ICD shocks [5] and social support [22,23,24]. In a second step, we applied the PROCESS regression path analysis modeling tool for SPSS to identify indirect mediating effects of PAs and NAs between fear of COVID-19 as an independent variable and depression (PHQ-8 score ≥ 10), anxiety (GAD-7 score ≥ 10) and PTSD (PDS score ≥ 14) as dependent variables, respectively. The significance level (two-sided *p*-value) was set at *p* < 0.05 and adjusted for multiple comparisons by post hoc chi-square testing.

## 3. Results

### 3.1. Description of the Study Sample

Sociodemographic and clinical characteristics of the study sample and the amount of missing data for each variable are depicted in Table 1 and Table 2. Of the 363 included participants, the majority (74.38%) were men who were significantly older, worked significantly more often in full-time jobs, had already more often retired and had significantly more myocardial infarctions in the past than women. On the other hand, women worked significantly more often in part-time jobs and had significantly more often an educational status lower than completed apprenticeship than men. The other variables did not differ significantly between the sexes. Both women and men had, on average, at least one ICD shock in the past, and almost one-third of all participants had a history of COVID-19.

### 3.2. Fear of COVID-19, Anxiety, Depression and PTSD in the Study Sample

Table 3 illustrates median and mean scores of the level of fear of COVID-19, anxiety, depression and PTSS, as well as the prevalence of GAD, MDD and PTSD according to established cut-off scores (see methods). Overall, women showed significantly higher levels of fear of COVID-19, of anxiety and of PTSS than men. Moreover, levels of depression did not differ statistically significantly between women and men. Although women indicated a trend toward a higher prevalence of GAD and PTSD than men did, the prevalence of GAD, MDD and PTSD did not differ significantly between women and men.

Mean and median scores for the individual items of the FCV-19S indicated rather low levels of fear of COVID-19 regarding the study sample in general (see Table 4). Items concerning physical stress responses due to the COVID-19 pandemic, such as palpitations, insomnia and sweating hands, were reported much less frequently than emotional or psychosocial concerns about the pandemic, including losing one’s life because of the virus. On a group level, women had significantly higher mean scores concerning items 2 and 5 than men.

Sociodemographics, clinical characteristics and the level of fear of COVID-19, anxiety, depression and PTSS are compared in Table 5 and Table 6. Levels of anxiety, depression and PTSS were significantly higher in younger than older patients. We also observed statistically significant differences in depression levels depending on educational status. Unemployed participants showed significantly higher levels of fear of COVID-19, anxiety, depression and PTSS. Participants who experienced ICD shocks in the past tended to have significantly higher levels of PTSS than those who did not experience any ICD shocks in the past. Interestingly, participants who reported a history of COVID-19 had significantly lower levels of fear of COVID-19 than those without a history of COVID-19. The other examined variables did not differ significantly regarding levels of fear of COVID-19, anxiety, depression and PTSS.

### 3.3. Identification of Predictors for Anxiety, Depression and PTSD

Table 7 highlights the eight independent variables from the binary multivariable logistic regression analysis for each of the dependent variables, a GAD-7 ≥ 10, a PHQ-8 ≥ 10, and a PDS ≥ 14, respectively. Omnibus tests indicated statistically significant regression models (*p* < 0.001). The regression models explained a variance (Nagelkerke R^2^) ranging from 26% to 31% (see Table 7). Due to the significantly higher levels of fear of COVID-19, anxiety, depression and PTSS in unemployed participants compared to participants with another work status, we decided to also include the variable “unemployed” (yes or no) as an independent variable in our regression models. Collinearity statistics showed no evidence of multicollinearity. The variance inflation factor (VIF) and tolerance for each independent variable were below, respectively, above the values suggested in literature (VIF < 10 and tolerance > 0.1 [25]).

Younger participants were significantly more likely to suffer from GAD or PTSD than older participants. Participants who received more social support were significantly less likely to have GAD, MDD or PTSD. A higher level of fear of COVID-19 was a significant predictor for GAD, MDD or PTSD. A history of COVID-19 significantly increased the odds of MDD. Participants who were unemployed demonstrated the highest odds ratios (OR), ranging from 6.54 for MDD to 10.39 for GAD. Regarding PTSD, being unemployed narrowly missed achieving statistical significance (*p* = 0.050). Higher numbers of ICD shocks showed a trend toward increasing the OR of GAD, MDD and PTSD, but failed to reach statistical significance as well as sex and educational status.

Moreover, we conducted a second binary multivariable logistic regression analysis, adding three independent variables, which might be further key factors influencing the research outcome: (1) a history of COVID-19 of closely related persons, such as family, partner and friends, and the awareness of COVID-19, as measured by (2) feeling well-informed about COVID-19 by authorities (yes or no) and (3) the employer (yes or no). The addition of three independent variables did not affect collinearity results (VIF < 10 and tolerance > 0.1 for each variable). Each significant variable of the first regression model remained statistically significant in the second model. In the latter, of the three newly added variables, only “feeling well-informed about COVID-19 by authorities” significantly reduced the OR for having MDD (OR = 0.29, 95% CI = 0.11 to 0.76, *p* = 0.012; see Supplementary Table S1).

### 3.4. Mediation Models between Fear of COVID-19 and Anxiety, Depression and PTSD

Our mediation models, which included the eight independent variables of the logistic regression (Table 7), revealed that the relationship between the level of fear of COVID-19 and GAD is fully mediated by PAs (indirect effect (IE): 0.032, 95% CI: 0.006–0.087) and NA (IE: 0.097, 95% CI: 0.054–0.209). Furthermore, we observed a fully mediated relationship of PAs (IE: 0.038, 95% CI: 0.010–0.095) and NA (IE: 0.077, 95% CI: 0.042–0.151) between the level of fear of COVID-19 and MDD. Regarding the relationship between the level of fear of COVID-19 and PTSD, we found that PAs did not mediate this relationship (IE: 0.018, 95% CI: −0.0007–0.050). However, NA partially mediated the relationship between the level of fear of COVID-19 and PTSD (IE: 0.049, 95% CI: 0.020–0.107). Higher PA scores indicated a lower possibility of GAD and MDD, while higher NA scores were indicative of a higher possibility of GAD, MDD and PTSD.

## 4. Discussion

### 4.1. Main Findings of the Study

In this sample of ICD patients, the prevalence according to established cut-off scores for anxiety was 9.19%, for depression 10.85% and for PTSD 12.99%. This finding is in line with a systematic review reporting that at least 11% of ICD patients suffer from anxiety or depressive disorders [26]. However, another meta-analysis concluded that at least 22% of ICD patients have clinically relevant anxiety and at least 15% have clinically relevant depression, while PTSD was shown in 12% of all patients [6]. These results imply similar rates of anxiety, depression and PTSD compared to all cardiac patients and higher rates compared to the general population [6]. In the latter, the prevalence of anxiety was estimated to be 7% [27], of depression 7–13% [28] and of PTSD only 1–2% [29]. The COVID-19 pandemic as a major stressor led to an increased prevalence of anxiety, depression and PTSD in the general population, with studies indicating anxiety rates of 25%, depression rates of 23%, and PTSD rates of even 30% due to the pandemic [30,31,32]. Interestingly, as a novelty of this study, which investigated the effects of the COVID-19 pandemic on mental health in ICD patients, we observed no elevated rates of anxiety, depression or PTSD compared to pre-COVID-19 results (see [6,26]). Of note, another study exploring the prevalence of anxiety and depression before and during the pandemic in patients with pulmonary arterial hypertension (PAH) found no significant changes in anxiety or depression between baseline and follow-up [33]. This discrepancy between increased levels of anxiety, depression and PTSS in the general population and rather constant results in other populations, such as ICD and PAH patients, might be explained by the stable and intensive medical care of these vulnerable patient populations during the pandemic. For instance, telemedicine became increasingly popular during the COVID-19 pandemic and demonstrated to be a valuable measure to improve quality of health care and to maintain contact with health care providers, possibly providing a feeling of safety [34].

The identified constant prevalence of anxiety, depression and PTSD in ICD patients during the pandemic compared to findings before, are also reflected in rather low levels of fear of COVID-19 in our study sample. Indeed, the mean score for fear of COVID-19, as measured by the FCV-19S, in our participants of 12 was below the score identified in the general population (range mean 13 to 27 [11,14,35]) and in patients with PAH (mean 19 [11]). However, it is difficult to compare those findings, since times of evaluation, examined populations and other characteristics are different than ours. Other studies examining the level of fear of COVID-19 and the prevalence of anxiety, depression and PTSD during the pandemic mostly focused on specific time points, whereas our study comprises the whole course of the pandemic. Since the COVID-19 pandemic was characterized by consecutive waves of infection with increasing and decreasing infection rates, including factors of prolonged restrictions and isolation, but also measures of loosening those restrictions, we might have captured a broader picture of the pandemic. Still, up to 20% of our participants reported high levels of fear of COVID-19, mainly concerning emotional worries about the pandemic, while physical stress responses were scarcely present.

This finding indicates that research might want to focus on subgroups of ICD patients who are especially vulnerable to the mental health impact of the pandemic. In our study, women demonstrated significantly higher levels of fear of COVID-19, anxiety and PTSS, which is in line with previous studies [11,35,36,37]. Additionally, younger age predicted anxiety and PTSD in our ICD patients, which is also consistent with the existing literature [11,36,37,38]. Another observation was that patients who received less social support were significantly more likely to have anxiety, depression and PTSD. This finding might not be specific to the COVID-19 pandemic, since social support is associated with mental health and well-being in general [39]. Prior studies indicated that low social support might exacerbate mental and physical health problems [40], and lead to increased levels of depression and anxiety in ICD patients [41]. Certainly, our finding underscores the importance of social support to help patients cope with challenging times such as the COVID-19 pandemic. A prior study identified being unemployed as a predictor for poor physical health status in ICD patients [38]. In our study, we found that being unemployed is also predictive of the mental health status in ICD patients and is the highest predictor of anxiety (OR = 10.4) and depression (OR = 6.5), especially for those patients who might benefit from cardiac rehabilitation combined with behavioral interventions [38]. Educational status and the number of ICD shocks did not predict anxiety, depression or PTSD in ICD patients in our study. Findings in the past were ambiguous while some studies indicated low education and more ICD shocks to be significant predictors [6,38], while others did not [37].

Most interestingly, a history of COVID-19 was found in 30% of our participants and significantly predicted depression, which was confirmed by another study concerning the mental health impact of the pandemic on patients with PAH [11]. Furthermore, higher levels of fear of COVID-19 significantly predicted anxiety, depression and PTSD. Although levels of fear were rather low on a group level, there appears to be a vulnerable subgroup of ICD patients prone to the detrimental mental health impacts of the pandemic. The other identified predictors in our study may provide insights into individual profiles of those patients most vulnerable to the pandemic, but further research is urgently required. In accordance with another study [11], patients who had a history of COVID-19, showed significantly lower levels of fear of COVID-19, although levels of anxiety, depression and PTSS were higher than in those without a history of COVID-19. These contrasting results possibly reflect the relatively short-term stress response, measured by the FCV-19S, compared to prolonged symptoms of depression, anxiety and PTSS.

Overall, it is crucial to better understand the relationship between fear of COVID-19, anxiety, depression and PTSD, respectively, since those psychological problems not only affect the quality of life but also mortality in ICD patients [7]. Hence, we explored as a novel approach the indirect mediating effects of fear of COVID-19 on anxiety, depression and PTSD via PAs and NAs. It was assumed that individual fear of COVID-19, including emotional concerns about the pandemic, would lead to a change in PAs and NAs, and thus, result in anxiety, depression and PTSD, according to the tripartite model [13]. Actually, the results of the study outlined fully mediated relationships of PAs and NAs between fear of COVID-19 and anxiety or depression, while the relationship between fear of COVID and PTSD was at least partially mediated by NA. These findings are in line with our results of the individual items of the FCV-19S, which pointed to higher levels of emotional concerns rather than physical stress responses due to the pandemic. Previous studies identified associations between PAs and social activity, happiness, and enjoyable events, whereas NA were associated with stress, health problems and unpleasant events. PAs were found to be specifically related to depression (lower PAs), while NAs were highly associated with depression and anxiety [12].

### 4.2. Implications of the Study

Although most patients seem to adapt well to their ICD and the COVID-19 pandemic, vulnerable ICD subgroups need to be identified, so that therapists can offer tailored interventions, such as supervised ICD patient groups [42] or gratitude groups [43]. Therapy in those patients might want to focus on the different aspects of PAs and NAs, to increase enjoyable PAs, such as being alert, determined, excited, or active, and to reduce unpleasable NA, such as being distressed, upset, scared, or feeling guilty. Prior studies highlighted the increase in PAs, and the decrease in NAs and depression in gratitude interventions [43]. Further interventions should be grounded in an extensive evaluation of different aspects leading to psychological problems, such as low social support, being unemployed and a history of COVID-19. Of note, our finding that patients who felt well-informed about COVID-19 by the authorities were significantly less likely to be depressed highlights the importance of educational interventions. These interventions should aim at raising awareness about COVID-19 in ICD patients, and counter misinformation, which might lead to increased levels of depression.

### 4.3. Limitations and Future Research

Our study has several strengths, such as the large sample size, considering the whole course of the COVID-19 pandemic, and using distinct instruments measuring fear of COVID-19, anxiety, depression, PTSD, social support and PAs and NAs. There are, however, some limitations to this study. First, our study was limited to a single centre, which restricts generalizability. Second, the majority of our participants were male (75%). Future studies might want to focus on female participants. Third, we unfortunately had no information regarding the vaccination status of our participants, which might be another important variable for analyzing the mental health impact of the COVID-19 pandemic on ICD patients [11]. Fourth, other studies focused on specific time points during and not the whole course of the pandemic, which comprises comparability. Additionally, longitudinal mental health data will be required to assess the long-term effects of the COVID-19 pandemic in ICD patients. We did not have any information, whether some of our participants had a psychiatric diagnosis of anxiety, depression, or PTSD already before the pandemic, which is another limitation. Finally, we have to critically point out that clinically relevant symptoms based on a cut-off score are not identical with a psychiatric diagnosis based on a clinical interview [44], which we did not conduct. Prospective studies should consider exploring individual profiles of ICD patients to identify subgroups most vulnerable to the pandemic. Novel approaches, such as a latent class analysis, might be suited for the identification of specific subgroups [45]. Although a history of COVID-19 of closely related persons, such as family, friends and partners, did not predict anxiety, depression or PTSD in ICD patients, future research might want to extend analyses regarding further COVID-19-related variables, which might be linked to those psychiatric symptoms or disorders. For instance, language competency might be vital for understanding COVID-19-related information and reduce psychological problems. In our study, we only included patients who were fluent in written and spoken German, but future studies could strive to include patients who are not fluent in the local language.

## 5. Conclusions

This study identified novel predictors for anxiety, depression and PTSD in ICD patients during the COVID-19 pandemic, including COVID-19-related variables. The detection of those predictors and additional indirect mediating effects of fear of COVID-19 on anxiety, depression and PTSD via PAs and NAs could help improve interventions for ICD patients most vulnerable to the pandemic, especially those with low social support, younger age, fear and a history of COVID-19, and those being unemployed. Patients with an ICD might also benefit from our findings during potential prospective pandemics. Future research might want to focus on the identification of vulnerable ICD subgroups.

## Figures and Tables

**Table 1:** Description of the study sample—sociodemographic characteristics.

	Overall (*n* = 363)	Women (*n* = 93)	Men (*n* = 270)	*p*-Value (*p* < 0.05) Group Analysis	*p*-Value Subgroup Analysis
Age (y), mean (SD)	57.96 (13.78)	52.04 (14.76)	59.99 (12.83)	**<0.001**	
Educational status	*n* = 358 (%)	*n* = 92	*n* = 266	**0.007**	*p* < 0.00625
Lower than completed apprenticeship or equivalent	14 (3.91)	9 (9.78)	5 (1.87)		**<0.001**
Completed apprenticeship or equivalent	205 (57.26)	49 (53.26)	155 (58.27)		0.402
High-school diploma or equivalent	52 (14.52)	16 (17.39)	36 (13.53)		0.365
University degree	88 (24.58)	18 (19.56)	70 (26.31)		0.194
Civil status	*n* = 358 (%)	*n* = 92	*n* = 266	0.246	*p* < 0.00625
Married	227 (63.40)	53 (57.60)	173 (65.03)		0.203
Divorced	54 (15.08)	19 (20.65)	35 (13.15)		0.083
Widowed	14 (3.91)	5 (5.43)	9 (3.38)		0.381
Single	64 (17.87)	15 (16.30)	49 (18.42)		0.647
Work status	*n* = 358 (%)	*n* = 92	*n* = 266	**<0.001**	*p* < 0.00625
Full time	99 (27.65)	12 (13.04)	87 (32.70)		**<0.001**
Part time	66 (18.43)	37 (40.21)	29 (10.90)		**<0.001**
Unemployed	41 (11.45)	16 (17.39)	24 (9.02)		0.028
Retired	153 (42.73)	27 (29.34)	126 (47.36)		**0.002**

Note: *n*: number. y: years. SD: standard deviation. Significant *p*-values are marked bold. *p*-values are based on chi-square test or Fisher’s exact test (if any cell number was <5) for categorical variables, and an unpaired *t*-test for continuous variables.

**Table 2:** Description of the study sample—clinical characteristics.

	Overall (*n* = 363)	Women (*n* = 93)	Men (*n* = 270)	*p*-Value (*p* < 0.05)
Smoking	*n* = 357 (%) 42 (11.76)	*n* = 92 9 (9.78)	*n* = 265 33 (12.45)	0.493
Past myocardial infarction	*n* = 351 (%) 137 (39.03)	*n* = 88 14 (15.90)	*n* = 263 123 (46.76)	**<0.001**
Number of ICD shocks in the past, mean (SD)	*n* = 336 1.14 (5.44)	*n* = 86 1.28 (3.78)	*n* = 250 1.10 (5.92)	0.782
History of COVID-19	*n* = 228 (%) 67 (29.38)	*n* = 59 18 (30.50)	*n* = 169 49 (28.99)	0.826

Note: *n*: number. y: years. ICD: implantable cardioverter defibrillator. SD: standard deviation. Significant *p*-values are marked bold. *p*-values are based on chi-square test or Fisher’s exact test (if any cell number was <5) for categorical variables, and an unpaired *t*-test for continuous variables.

**Table 3:** Median and mean scores of levels of fear of COVID-19, anxiety, depression and post-traumatic stress symptoms (PTSS) and disorders (PTSD) in the study sample.

	Overall; Median (Range) or Mean (SD)	Women	Men	*p*-Value (*p* < 0.05)
Level of fear of COVID-19 (FCV-19S)	*n* = 357 11 (7–35) 11.94 (4.85)	*n* = 90 12 (7–28) 12.88 (5.21)	*n* = 267 10 (7–35) 11.64 (4.68)	**0.036**
GAD-7	*n* = 348	*n* = 91	*n* = 257	
	2 (0–21)	3 (0–19)	1 (0–21)	
	3.29 (4.19)	4.04 (4.12)	3.02 (4.20)	**0.048**
GAD-7 score ≥ 10, *n* (%)	32 (9.19)	11 (12.08)	21 (8.17)	0.266
PHQ-8	*n* = 350	*n* = 87	*n* = 263	
	4 (0–23)	5 (0–17)	3 (0–23)	
	4.53 (4.03)	5.14 (3.66)	4.34 (4.14)	0.115
PHQ-8 score ≥ 10, *n* (%)	38 (10.85)	9 (10.34)	29 (11.02)	0.859
PDS	*n* = 354	*n* = 91	*n* = 263	
	2 (0–41)	5 (0–41)	2 (0–40)	
	5.60 (8.09)	7.95 (9.25)	4.78 (7.50)	**0.004**
PDS score ≥ 14, *n* (%)	46 (12.99)	16 (17.58)	30 (11.40)	0.131

Note: FCV-19S: Fear of COVID-19 Scale. GAD-7: Generalized Anxiety Disorder-7. PHQ-8: Patient Health Questionnaire-8. PDS: Post-Traumatic Stress Diagnostic Scale. Significant *p*-values are marked bold. *p*-values are based on chi-square test for categorical variables, and an unpaired *t*-test for continuous variables.

**Table 4:** Individual item results for the FCV-19S.

FCV-19S Items	Overall (*n* = 357); Median (Range) or Mean (SD)	Women (*n* = 90)	Men (*n* = 267)	*p*-Value (*p* < 0.05)
**1** I am most afraid of coronavirus-19	2 (1–5) 2.34 (1.19)	2 (1–5) 2.39 (1.20)	2 (1–5) 2.00 (1.18)	0.652
*score ≥ 4 (agree or strongly agree), n (%)*	71 (19.88)	18 (20.0)	53 (19.85)	
**2** It makes me uncomfortable to think about coronavirus-19	2 (1–5) 2.18 (1.17)	2 (1–5) 2.41 (1.19)	2 (1–5) 2.11 (1.15)	**0.032**
*score ≥ 4, n (%)*	63 (17.64)	20 (22.22)	43 (16.10)	
**3** My hands become clammy when I think about coronavirus-19	1 (1–5) 1.25 (1.09)	1 (1–4) 1.32 (0.71)	1 (1–5) 1.23 (0.63)	0.291
*score ≥ 4, n (%)*	5 (1.40)	2 (2.22)	3 (1.12)	
**4** I am afraid of losing my life because of coronavirus-19	2 (1–5) 1.88 (1.09)	2 (1–5) 2.02 (1.10)	1 (1–5) 1.83 (1.09)	0.162
*score ≥ 4, n (%)*	32 (8.96)	8 (8.88)	24 (8.98)	
**5** When watching news and stories about coronavirus-19 on social media, I become nervous or anxious	1 (1–5) 1.74 (1.01)	2 (1–5) 1.96 (1.07)	1 (1–5) 1.67 (0.99)	**0.023**
*score ≥ 4, n (%)*	31 (8.68)	11 (12.22)	20 (7.49)	
**6** I cannot sleep because I am worrying about getting coronavirus-19	1 (1–5) 1.29 (0.68)	1 (1–5) 1.41 (0.87)	1 (1–5) 1.25 (0.61)	0.102
*score ≥ 4, n (%)*	9 (2.52)	5 (5.55)	4 (1.49)	
**7** My heart races or palpitates when I think about getting coronavirus-19	1 (1–5) 1.30 (0.67)	1 (1–4) 1.39 (0.71)	1 (1–5) 1.27 (0.66)	0.156
*score ≥ 4, n (%)*	7 (1.96)	2 (2.22)	5 (1.87)	

Note: *p*-values are based on an unpaired *t*-test for continuous variables. Significant *p*-values are marked bold.

**Table 5:** Comparison between sociodemographic characteristics and levels of fear of COVID-19, anxiety, depression and PTSS.

	FCV-19S; Mean (SD)	GAD-7	PHQ-8	PDS
Age		*p* ***	*p* **	*p* ***
Age < 65 years	12.29 (5.06)	4.05 (4.64)	4.96 (4.35)	6.69 (9.15)
≥65 years	11.36 (4.37)	1.87 (2.77)	3.73 (3.28)	3.60 (5.24)
Educational status			*p* *	
Lower than completed apprenticeship or equivalent	12.15 (6.12)	4.35 (5.95)	4.92 (4.60)	7.71 (10.63)
Completed apprenticeship or equivalent	12.17 (5.27)	3.26 (4.04)	4.35 (3.73)	5.39 (8.02)
High-school diploma or equivalent	12.73 (4.49)	3.78 (5.08)	6.18 (5.52)	7.79 (9.50)
University degree	11.02 (3.68)	2.96 (3.62)	3.90 (3.32)	4.55 (6.72)
Civil status				
Married	11.85 (4.70)	3.04 (3.81)	4.29 (3.86)	4.70 (6.91)
Divorced	13.15 (5.27)	3.86 (4.88)	5.37 (4.56)	7.61 (9.91)
Widowed	12.07 (5.34)	2.15 (3.13)	4.50 (3.68)	5.23 (7.99)
Single	11.27 (4.84)	3.51 (4.75)	4.51 (4.19)	6.53 (9.44)
Work status	*p* ***	*p* ***	*p* ***	*p* ***
Full time	10.74 (3.48)	2.78 (3.39)	3.68 (3.39)	4.04 (5.83)
Part time	12.34 (4.60)	3.59 (3.61)	4.79 (3.14)	5.94 (8.35)
Unemployed	15.03 (6.53)	6.51 (6.78)	7.23 (6.31)	10.58 (13.33)
Retired	11.70 (4.87)	2.60 (3.57)	4.27 (3.75)	5.08 (6.86)

Note: *p*-values are based on an unpaired *t*-test and one-way ANOVA for continuous variables. *p* < 0.05: *. *p* < 0.01: **. *p* < 0.001: ***.

**Table 6:** Comparison between clinical characteristics and levels of fear of COVID-19, anxiety, depression and PTSS.

	FCV-19S; Mean (SD)	GAD-7	PHQ-8	PDS
Smoking				
yes	12.69 (5.68)	3.90 (4.77)	5.29 (4.91)	6.51 (9.10)
no	11.88 (4.74)	3.19 (4.00)	4.38 (3.82)	5.41 (7.89)
Past myocardial infarction				
yes	12.12 (5.08)	3.26 (4.18)	4.68 (4.13)	5.33 (8.24)
no	11.80 (4.69)	3.25 (4.16)	4.47 (4.01)	5.79 (8.02)
ICD shocks				*p* *
yes	12.30 (5.69)	3.75 (4.24)	4.69 (3.91)	7.21 (9.23)
no	11.81 (4.49)	3.09 (4.17)	4.45 (4.10)	4.89 (7.48)
History of COVID-19	*p* *			
yes	10.70 (4.29)	4.22 (4.97)	5.60 (4.68)	6.61 (8.62)
no	12.38 (4.92)	3.20 (3.77)	4.61 (3.65)	5.89 (8.13)

Note: *p*-values are based on an unpaired *t*-test for continuous variables. *p* < 0.05: *.

**Table 7:** Binary logistic regression for GAD (GAD-7 ≥ 10), MDD (PHQ-8 ≥ 10) and PTSD (PDS ≥ 14) in patients with an ICD.

	GAD		MDD		PTSD	
Variables	OR (95% CI)	*p*-Value	OR (95% CI)	*p*-Value	OR (95% CI)	*p*-Value
Male sex	0.74 (0.27 to 1.97)	0.548	0.55 (0.20 to 1.49)	0.242	0.99 (0.42 to 2.34)	0.990
Age	0.95 (0.91 to 0.99)	**0.017**	0.97 (0.93 to 1.01)	0.277	0.92 (0.88 to 0.95)	**<0.001**
Higher educational status	0.68 (0.41 to 1.15)	0.154	1.05 (0.67 to 1.64)	0.831	0.78 (0.51 to 1.21)	0.279
Social support	0.91 (0.85 to 0.98)	**0.017**	0.88 (0.83 to 0.94)	**<0.001**	0.91 (0.85 to 0.97)	**0.003**
ICD shock number	1.04 (0.99 to 1.10)	0.070	1.04 (0.99 to 1.09)	0.077	1.05 (0.99 to 1.10)	0.068
History of COVID-19	1.72 (0.51 to 5.75)	0.374	3.58 (1.31 to 9.74)	**0.012**	1.65 (0.59 to 4.60)	0.335
Level of fear of COVID-19	1.10 (1.03 to 1.19)	**0.005**	1.12 (1.04 to 1.21)	**0.001**	1.14 (1.07 to 1.23)	**<0.001**
Being unemployed	10.39 (2.37 to 46.25)	**0.002**	6.54 (1.72 to 24.88)	**0.006**	3.62 (0.99 to 13.15)	0.050
Nagelkerke R²	0.28		0.26		0.31	

Note: OR: odds ratio. CI: confidence interval. MDD: major depressive disorder. Significant *p*-values are marked bold.

## Data Availability

The data presented in this study are available on request from the corresponding author.

## Supplementary Materials

The following supporting information can be downloaded at: https://www.mdpi.com/article/10.3390/jcm12216884/s1, Table S1. Binary logistic regression for GAD (GAD-7 ≥ 10), MDD (PHQ-8 ≥ 10) and PTSD (PDS ≥ 14) in patients with an ICD—inclusion of further COVID-19 related variables.
