# Supplementary material for: Fear of COVID-19 Predicts Depression, Anxiety and Post-Traumatic Stress Disorders in Patients with Implantable Cardioverter Defibrillators and Is Mediated by Positive and Negative Affects—A Cross-Sectional Study
Source: J Clin Med. 2023 Oct 31;12(21):6884. doi: 10.3390/jcm12216884 (PMC10648893; doi:10.3390/jcm12216884)
Supplement: Supplementary file 1 [file jcm-12-06884-s001.zip › jcm-2681471-supplementary.pdf]

Table S1. Binary logistic regression for GAD (GAD-7 ≥10), MDD (PHQ-8 ≥10) and PTSD (PDS ≥14) in patients with an ICD – inclusion of further COVID-19 related variables.

	GAD		MDD		PTSD	
**Variables**	**OR (95% CI)**	**p-value**	**OR (95% CI)**	**p-value**	**OR (95% CI)**	**p-value**
Male sex	0.77 (0.28 to 2.12)	0.614	0.67 (0.24 to 1.90)	0.457	1.06 (0.44 to 2.53)	0.894
Age	0.94 (0.91 to 0.98)	**0.013**	0.97 (0.93 to 1.01)	0.169	0.92 (0.88 to 0.95)	**<0.001**
Higher educational status	0.67 (0.39 to 1.14)	0.148	1.01 (0.62 to 1.62)	0.965	0.76 (0.49 to 1.18)	0.228
Social support	0.91 (0.85 to 0.98)	**0.016**	0.88 (0.83 to 0.94)	**<0.001**	0.90 (0.85 to 0.96)	**0.003**
ICD shock number	1.04 (0.99 to 1.10)	0.074	1.04 (0.99 to 1.10)	0.079	1.05 (0.99 to 1.10)	0.066
History of COVID-19	1.52 (0.43 to 5.34)	0.507	2.87 (1.01 to 8.16)	**0.047**	1.82 (0.63 to 5.29)	0.266
Level of fear of COVID-19	1.11 (1.03 to 1.19)	**0.005**	1.13 (1.04 to 1.21)	**0.001**	1.15 (1.07 to 1.23)	**<0.001**
Being unemployed	10.18 (2.22 to 46.62)	**0.003**	5.21 (1.30 to 20.76)	**0.019**	3.36 (0.88 to 12.78)	0.075
History of COVID-19 of a closely related person	1.88 (0.38 to 9.31)	0.436	2.63 (0.53 to 12.87)	0.233	0.75 (0.23 to 2.46)	0.644
Feeling well-informed about COVID-19 by authorities	0.49 (0.16 to 1.51)	0.217	0.29 (0.11 to 0.76)	**0.012**	0.46 (0.18 to 1.19)	0.113
Feeling well-informed about COVID-19 by employer	1.13 (0.28 to 4.61)	0.855	0.48 (0.15 to 1.52)	0.219	0.91 (0.27 to 3.09)	0.886
Nagelkerke R^2	0.29		0.31		0.33	

Note: OR: odds ratio. CI: confidence interval. MDD: major depressive disorder. Significant p-values are marked bold.
